# MicroRNA-129-1-3p Represses the Progression of Triple-Negative Breast Cancer by Targeting the GRIN2D Gene

**DOI:** 10.1155/2022/1549357

**Published:** 2022-03-07

**Authors:** Qi Li, Zehui Gu, Qi Tan, Liqun Ren, Suxian Chen

**Affiliations:** ^1^Department of Pathology, The Third Affiliated Hospital of Jinzhou Medical University, No. 5-2 Heping Road, Jinzhou, Liaoning 120001, China; ^2^Department of Experimental Pharmacology and Toxicology, School of Pharmacy, Jilin University, 1266 Fujin Road, Changchun, Jilin 130021, China

## Abstract

The aberrant expression of miRNA is strongly linked to numerous stages of triple-negative breast cancer (TNBC) progression, and it plays an indispensable role in the process from tumor onset and progress to invasion and metastasis. In this study, we first transfected miR-129-1-3p mimics and inhibitor into MDA-MB-231 TNBC cells, respectively. Then, we assessed the pathological role of miR-129-1-3p in MDA-MB-231 cells. The results showed that miR-129-1-3p were successfully inserted into MDA-MB-231 cells. Besides, miR-129-1-3p could distinctively repress the growth, migration along with infiltration of MDA-MB-231 cells, which might be related to the inhibition of GRIN2D expression. Our results indicate that miR-129-1-3p was illustrated to serve as a tumor repressor via targeting GRIN2D in TNBC cells and highlight that the restoration of miR-129-1-3p might be a new treatment target for TNBC.

## 1. Introduction

TNBC (triple-negative breast cancer) is an aggressive subclass of breast cancer (BC), representing around 10–20% of all invasive BCs [1, 2]. As TNBC does not respond to endocrine treatment and always shows low effectiveness to targeted therapy, chemotherapy is still the most effective method of preventing cancer cell growth along with metastasis [3]. Nevertheless, because of the adverse side effects of chemotherapy, research into new safe and effective drugs or methods for TNBC is continuing.

The importance of milk calcium in newborns' growth and development requires that the mammary glands are intrinsically connected with Ca^2+^ influx. The expression of some calcium channels and calcium pumps that transport Ca^2+^ into milk will change in some BCs [4]. The calcium channels and calcium pumps may be specific to different molecular subtypes of BC [5]. Research evidence illustrates that Ca^2+^ influx has a vital role in cellular growth, migration, angiogenesis, and multidrug resistance for BC [6–11].

Investigations have documented that miRNAs may play diverse roles in different kinds of cancers, with some acting as tumor repressor genes and others as oncogenes, depending on the pathway or gene they modulate [12–14]. miR-129-1-3p is one of the mature miR-129 family members, which plays an indispensable role in tumor onset and progress [15]. It is also known that miR-129-1-3p can interfere with tumor occurrence and prognosis through acting on diverse molecular targets in serous ovarian cancer, gastric cancer, as well as non-small cell lung cancer [16–18].

miR-129-1-3p role in TNBC biology remains unclear. Hence, we purposed to study the mediating role of miRNA-129-1-3p in repressing the growth, migration, as well as infiltration of TNBC cells to supply evidence for the development of new medication for the disease.

## 2. Materials and Methods

The details of the reagents and materials used in this study are in Appendix I.

### 2.1. Cell Culture and miR-129-1-3p Mimics and Inhibitors

MDA-MB-231 human TNBC cells were supplied by the Shanghai Institute of Cell Biology (Shanghai, China). The cells were inoculated in RPMI (SH30243.01, Hyclone, United States) enriched with 10% FBS (SH30084.03, Hyclone, United States) along with penicillin-streptomycin under 37°C and 5% CO_2_ conditions.

Mimics of miR-129-1-3p along with suppressors were prepared to examine miR-129-1-3p influence on cells. miR-129-1-3p mimics were synthesized by Jiangsu GenePharma Company (Jiangsu, China). Oligonucleotide sequences consisted of the following:

- Mimics of has-miR-129-1-3p: AAGCCCUUACCCCAAAAAGUAU
- has-miR-129-1-3p suppressor: AUACUUUUUGGGGUAAGGGCUU
- Mimics of miRNA single-strand negative control (mimics NC): UUGUACUACACAAAAGUACUG
- miRNA inhibitors single-strand negative control (inhibitors NC): CAGUACUUUUGUGUACAA

### 2.2. qRT-PCR Assays

qRT-PCR was adopted to assess miR-129-1-3p content in all experimental tissues. The details of the kits used during the qRT-PCR are in Appendix I. The sequences of primers used herein were as follows:

- GRIN2D forward 5 ′-TTCATCTTCGAGTACCTCAG-3′
- GRIN2D reverse 5′-CACCGAATTATTGAACACCA-3′
- GAPDH forward 5′-CATGAGAAGTATGACAACAGCCT-3′
- GAPDH reverse 5′-AGTCCTTCCACGATACCAAAGT-3′
- miRNA-129-1-3p forward 5′-GCCCTTACCCCAAAAAGTATAAA-3′
- miRNA-129-1-3p reverse 5′-CCAGTCTCAGGGTCCGAGGTATTC-3′
- U6 forward 5′-GCTTCGGCAGCACATATACTAAAAT-3′
- U6 reverse 5′-CGCTTCACGAATTTGCGTGTCAT-3′

### 2.3. Cell Counting CCK-8 Assay and Colony Formation Assay

1 × 10^4^ MDA-MB-231 cell transfects/well were inoculated into 96-well plates. CCK-8 solution in 10 *μ*L was introduced to every well after incubation for 24 hours. After that, a microplate reader (MK3, Thermo Fisher Scientific, United States) was employed to determine the absorbance after incubation under 37°C and 5% CO_2_ conditions for 2 hours, and the cell survival rate and the cell repression percentages were then calculated [19].

#### 2.3.1. Colony Formation Assay

Cell clone formation assay is an important technique used to assay for cell growth. We inoculated transfected MDA-MB-231 into 6-well plates (5 × 10^2^ cells/well), while changing the growth medium every three days, and observations were performed every day. We used an enzyme-linked spot image automatic analyzer to analyze, scan, and take pictures. Afterwards, CFE (clone formation rate) was then computed as follows: CFE = average amount of clones/number of inoculated cells × 100%.

### 2.4. Scratch Assay and Transwell Migration and Invasion Assay

#### 2.4.1. Scratch Assay

To ensure that the fusion rate of transfected cells in the 6-well plate reached 100%, 1 × 10^6^ cells/mL were introduced to each well. After the cells adhered to the wall, a line was drawn evenly with a 10 *μ*L spearhead, about every 0.5~1.0 cm, and was evaluated by measuring the width of scratches using ImageJ software.

#### 2.4.2. Transwell Migration and Invasion Assay

24-well Transwell (8 *μ*m pore size) uncoated with Matrigel was used to perform the cell migration assays. We suspended 1 × 10^5^ cells in total in the two chambers: 500 *μ*l RPMI 1640 enriched with 1% FBS in the upper compartment and 750 *μ*l RPMI 1640 enriched with 20% FBS in the lower chamber. Matrigel along with the cells remaining in the upper compartment was swabbed using cotton swabs after 24 hours of incubation. Meanwhile, we fixed the cells on the membrane's lower surface in 4% PFA and then stained them with 0.5% crystal violet. Subsequently, we observed the cells (at ×100 magnification) and determined the numbers under microscope (DMI6000B, Leica, Germany).

### 2.5. Calcium Levels Assay and Western Blot

#### 2.5.1. Calcium Levels Assay

The cells were inoculated with a serum-free medium enriched with 0.5–5 *μ*M Fluo-3 AM at 20–37°C for 15–60 minutes, the fluorescent probe was loaded, and the cells were rinsed once or twice with PBS. After washing, the solution was incubated for another 20–30 minutes to ensure that Fluo-3 AM was wholly transformed into Fluo in the cells. DAPI solution (C1005, Beyotime, China) was introduced, and then subsequently incubated at RT (room temperature) for five minutes. We rinsed the cells in PBS twice, and then observed with a fluorescence microscope, and pictures were taken. The expression rate of Ca^2+^ positive cells was calculated as Ca^2+^ positive cells in the field divided by all cells in the field ×100%.

#### 2.5.2. Western Blot

The cell proteins were extracted with cell lysate, and the protein concentration detected. SDS-PAGE separation gel and SDS-PAGE laminated gel were prepared according to the required molecular weights of the proteins. The gel was extracted by electrophoresis and membrane transfer and sealed, and then the primary antibody was introduced and inoculated overnight at 4°C or at 25°C for 2 hours, and the secondary antibody was incubated at 25°C for 2 hours. The PVDF film was taken out, dropwise ECL chemiluminescence super-sensitive color reagent (A liquid: B liquid =1 : 1, P0018FM, Beyotime, China) was added to it, and the film was placed in a cassette to expose and develop the photograph. The ImageJ software was adopted to analyze the gray value of each group of bands and do the statistics. The following primary antibodies were purchased: GRIN2D (ABclonal, China; 1 : 800), CD147 (R&D Systems, USA; 1 : 1000), Matrix metallopeptidase 2 (MMP-2; Cell Signaling Technology, USA; 1 : 1000), Matrix metallopeptidase 9 (MMP-9; Cell Signaling Technology, USA; 1 : 1000), VEGF; (R&D Systems, USA; 1 : 1000), and GAPDH (ABclonal, China; 1 : 2000).

### 2.6. Data Analysis

The data were processed in the SPSS Statistics for Windows, V.17.0. Chicago: SPSS Inc. All results for continuous variables are given as mean ± standard deviation (SD). The *t*-test was implemented for comparison between the two groups, with *P* < .05 signifying statistical significance.

## 3. Results

### 3.1. miR-129-1-3p Intertargeted with GRIN2D in MDA-MB-231 Cells

**Figure 1:**
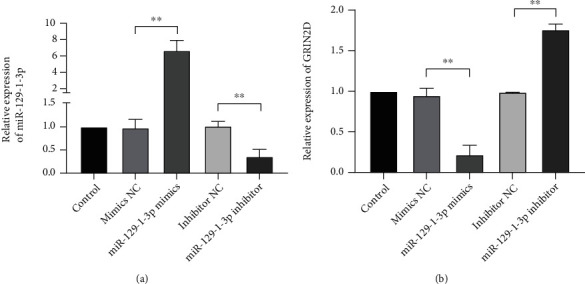
Comparison of the expression of (a) miR-129-1-3p and (b) GRIN2D mRNA in transfects of miR-129-1-3p mimic or inhibitor. RT-qPCR assessment of miR-129-1-3p and GRIN2D mRNA contents. Untransfected cells were used for comparison; ^∗∗^*P* < .01.

As shown in Figure 1(a), in contrast with the control group, mRNA levels of miR-129-1-3p along with GRIN2D in the NC groups were not remarkably different (*P* > .05). Relative to the mimics NCs, the content of miR-129-1-3p in the mimics was remarkably elevated (*P* < .01). Meanwhile, miR-129-1-3p levels in the miR-129-1-3p suppressor group was dramatically decreased in contrast with the suppressor NC group (*P* < .01), while GRIN2D mRNA level showed an opposite trend with miR-129-1-3p (Figure 1(b)).

### 3.2. miR-129-1-3p Repressed the Growth of MDA-MB-231 Cells

**Figure 2:**
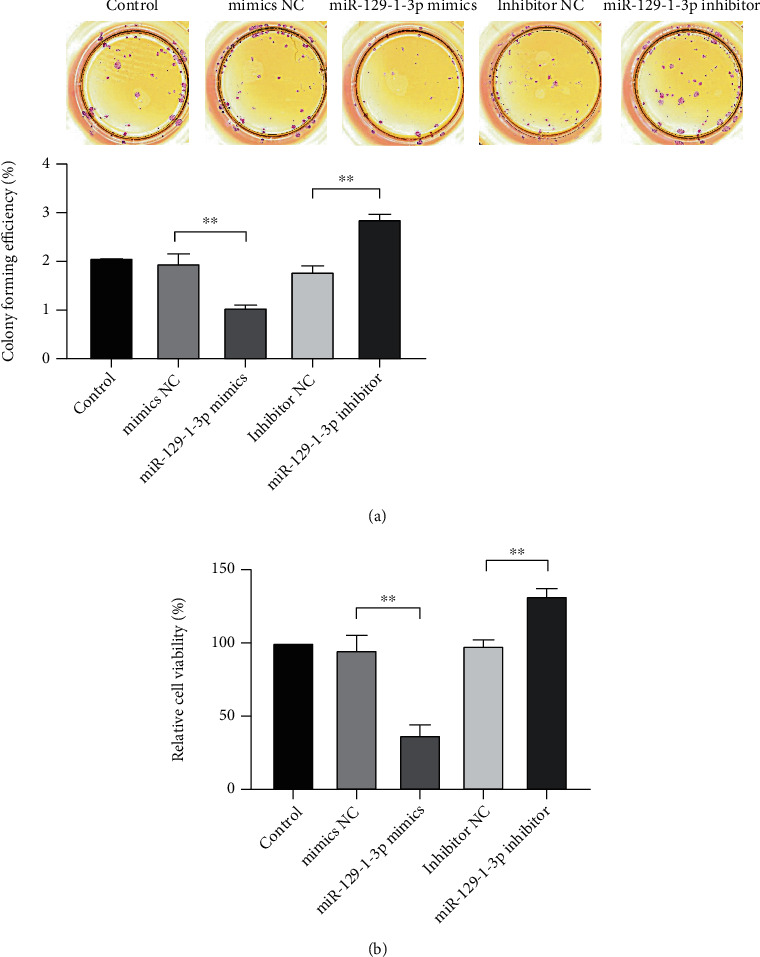
miR-129-1-3p repressed the growth of MDA-MB-231 cells. (a) Colony formation assay. Images illustrating the efficiency of colony forming (left lower). (b) CCK-8 assessment of cell growth (right lower); ^∗∗^*P* < .01.

As illustrated in Figure 2(a), in contrast to the controls, the clone formation ability of cells in the NC groups was not dramatically different (*P* > .05). The mimics of miR-129-1-3p dramatically diminished the clone formation rate of cells (*P* < .01) in contrast with the mimics NC. Besides, miR-129-1-3p inhibitor dramatically increased the rate of cell clone formation (*P* < .01) relative to the inhibitor NC.

As shown in Figure 2(b), the survival rate of MDA-MB-231 cell transfects of miR-129-1-3p mimics was dramatically reduced (*P* < .01) in contrast with that of the mimics NC cell transfects. The MDA-MB-231 cell transfects of miR-129-1-3p suppressor exhibited dramatically elevated the survival rate of cells than inhibitor NC cell transfects (*P* < .01).

### 3.3. miR-129-1-3p Dampened the Migration along with Infiltration of MDA-MB-231 Cells

**Figure 3:**
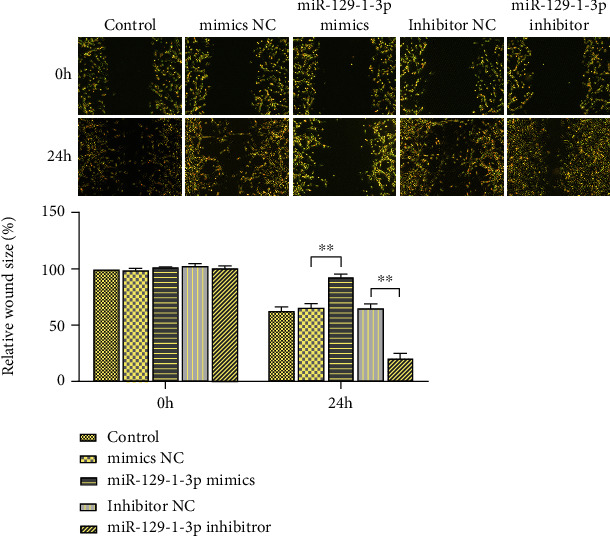
Scratch assays of miR-129-1-3p repression of MDA-MB-231 cell migration. Wound healing evaluation of the migration in MDA-MB-231 cells (×40 magnification); ^∗∗^*P* < .01.

As illustrated in Figure 3, in contrast with the controls, there was no remarkable difference in growth and migration ability between the mimics NC group and the inhibitor NC group (*P* > .05). Relative to mimics NC cell transfects, the repair area of the scratch area was remarkably decreased in miR-129-1-3p mimic cell transfects (*P* < .01). The miR-129-1-3p suppressor remarkably increased the repair area of the scratch area and fused the cells in contrast with inhibitor NC (*P* < .01).

**Figure 4:**
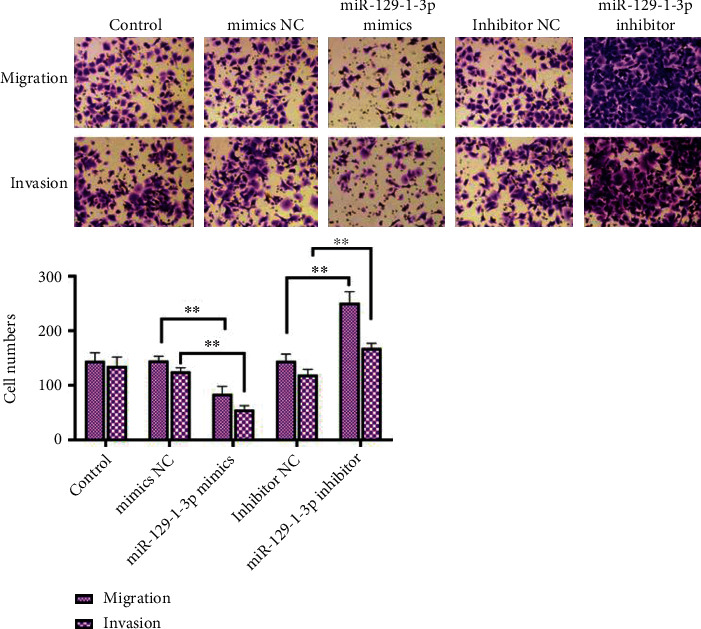
Transwell assays of miR-129-1-3p repression in MDA-MB-231 cells migration and infiltration. MDA-MB-231 BC cells migration was explored using transwell assay and observed with a microscope (×100 magnification); ^∗∗^*P* < .01.

MDA-MB-231 cells had vertical migration ability. As illustrated in Figure 4, the number of downmigration cells in the miR-129-1-3p mimic cell transfects was remarkably diminished than in the mimics NC cell transfects (*P* < .01). miR-129-1-3p inhibitor cell transfects exhibited remarkably increased number of downmigration cells relative to inhibitor NC transfects (*P* < .01).

As depicted in Figure 4, relative to the mimic NC cell transfects, the number of invading cells in miR-129-1-3p mimics was remarkably decreased (*P* < .01). In contrast with inhibitor NC, the miR-129-1-3p inhibitor group remarkably increased the number of cells descending through the matrix gel (*P* < .01).

**Figure 5:**
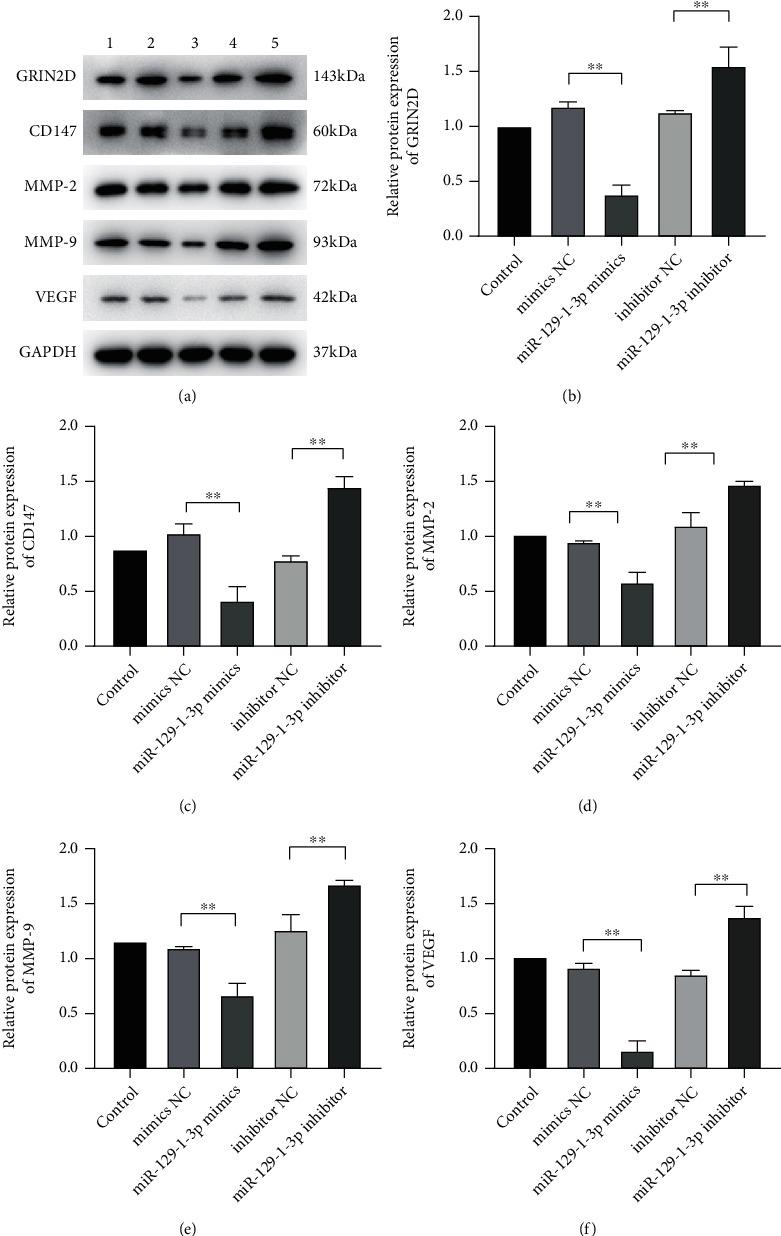
(a) Western blot electrophoresis development strip image. Protein expression of (b) GRIN2D, (c) CD147, (d) MMP-2, (e) MMP-9, and (f) VEFG were explored with western blotting. ^∗∗^*P* < .01.

Herein, we detected the expression of MMP-9, CD147, MMP-2, as well as VEGF protein linked to tumor cell migration along with infiltration in MDA-MB-231 cells via Western blot experiment. The results showed that miR-129-1-3p mimics reduced the expression of CD147, MMP-2, MMP-9, and VEGF protein in MDA-MB-231 cells, whereas miR-129-1-3p inhibitor had the opposite influence (Figure 5).

### 3.4. miR-129-1-3p Repressed the Content of Calcium in MDA-MB-231 Cells

**Figure 6:**
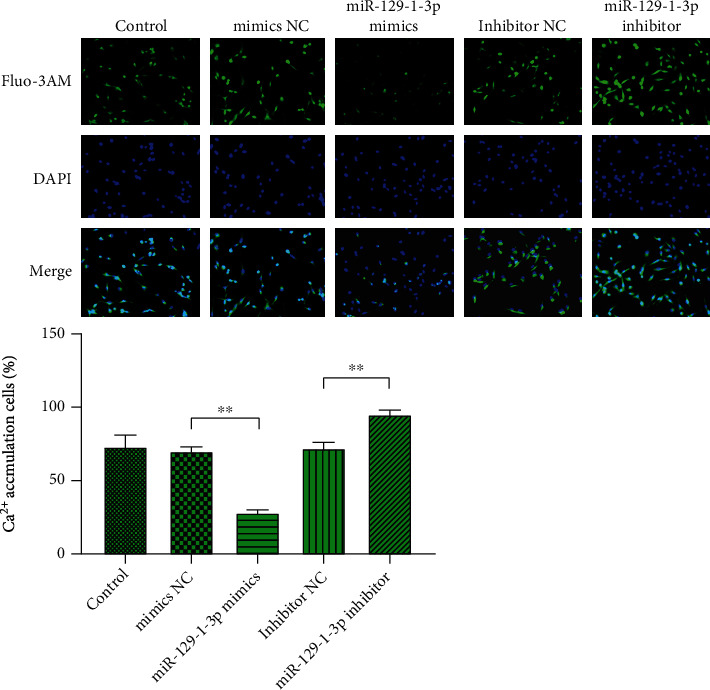
miR-129-1-3p represses MDA-MB-231 intracellular calcium content. Intracellular Ca^2+^ levels were explored via fluorescence imaging using Fluo-3 AM staining. Images illustrating Fluo-3 AM-positive cells (nether) (×100 magnification, upper) and percentages; ^∗∗^*P* < .01.

As illustrated in Figure 6, compared to controls, there was no remarkable difference in the Ca^2+^ positive cell expression rate in the NC transfects (*P* > .05). In contrast with the mimics NC cell transfects, the rate of expression of Ca^2+^ positive cells in the miR-129-1-3p mimic transfects was dramatically reduced (*P* < .01). The rate of expression of Ca^2+^ positive cells in the transfects of miR-129-1-3p inhibitor was remarkably increased (*P* < .01) in contrast to the inhibitor NC transfects. Furthermore, the protein contents of GRIN2D in the miR-129-1-3p mimic transfects were remarkably decreased (*P* < .01) than in the controls, while in the miR-129-1-3p suppressor transfects they were remarkably elevated (*P* < .01) (Figure 5).

## 4. Discussion

miRNA is involved in almost all aspects of cancer biology, including angiogenesis, drug resistance, apoptosis, proliferation, invasion, and metastasis. Studies have shown that miRNAs may act as tumor suppressors or oncogenes in many cancers, depending on which pathway or gene they regulate. The pathogenesis of triple-negative breast cancer is complicated, with many influencing factors, and the prognosis is not good. More and more studies have shown that miRNAs are involved in regulating the occurrence and progression of breast cancer. Among them, miR-129-1-3p has been confirmed to play an important role in tumorigenesis and development. However, whether miR-129-1-3p is involved in the occurrence and development of breast cancer has not yet been reported. Herein, we studied the function of miR-129-1-3p in TNBC biology. In transfected MDA-MB-231 cells, CCK-8 and plate cloning experiments illustrated that miR-129-1-3p mimics could repress the growth of MDA-MB-231 cells and the ability to form clones, whereas miR-129-1-3p inhibitors have the opposite influence. The results illustrate that miR-129-1-3p may play a particular role in repressing TNBC occurrence and development. A similar function was found in research for ovarian cancer. Another report documented that miR-129-1-3p along with miR-129-2-3p levels were remarkably reduced in serous ovarian cancer, and miR-129-1-3p overexpression could considerably dampen the growth of SKOV3 cells, which suggested that restoration of miR-129-1-3p expression may be a new treatment strategy for ovarian cancer [16].

Tumor invasion or metastasis is one of the most critical signs of malignant tumors and the most important fatal factor for BC [20]. Wang et al. illustrated that miR-129-1-3p represses the migration of gastric cancer cells BGC-823 [17]. Herein, we established that miR-129-1-3p mimics repressed the potential of MDA-MB-231 cells to migrate and invade far away, while miR-129-1-3p suppressor enhanced MDA-MB-231 cells' ability to migrate, as well as invade far away. It indicates that miR-129-1-3p dampens the migration along with infiltration of MDA-MB-231 cells.

CD147 is a highly glycosylated transmembrane protein. Many studies have shown that CD147 is involved in multiple vital steps in cancer development and is excessively expressed in human tumors such as BC and ovarian cancer [5, 21]. MMP-2 and MMP-9 have a strong ability to degrade the main components of the basement membrane. Various evidence indicate that MMP-2 along with MMP-9 are overexpressed in malignant tumors, including BC [22, 23]. Besides, MMP-2 coupled with MMP-9 was reported to be involved in the adhesion, migration, as well as infiltration of MDA-MB-231 cells [24].

Furthermore, MMP-2 and MMP-9 can remarkably affect angiogenesis in malignant tumors by activating VEGF [25]. The VEGF is a remarkably specific vascular endothelial cell growth factor promoting elevated vascular permeability, vascular endothelial cell migration, growth, and angiogenesis. It participates in the occurrence and progression of many angiogenesis-dependent diseases, including cancer [26]. In addition, the increase in VEGF levels is also linked to tumor formation and drug resistance [27]. Herein, we detected the expression of MMP-9, CD147, MMP-2, as well as VEGF protein linked to tumor cell migration along with infiltration in MDA-MB-231 cells via Western blot experiment. The results showed that miR-129-1-3p mimics reduced the expression of CD147, MMP-2, MMP-9, and VEGF protein in MDA-MB-231 cells, whereas miR-129-1-3p inhibitor had the opposite influence. The above results further verified the results of cell scratches and transwell experiments, suggesting that miR-129-1-3p has a vital role in repressing the migration and infiltration of MDA-MB-231 cells.

Ca^2+^ is also closely linked to BC cell growth [8], invasion and metastasis [9], angiogenesis [10], and multidrug resistance [11]. Kanwar et al. found in mouse BC metastasis model experiments that higher Ca^2+^ levels increase the survival and metastasis ability of BC tumor cells [28]. Liu et al. documented that calcium channel inhibitors (SOCC) can inhibit the migration of MCF-7 cells, indicating that abnormal Ca^2+^ levels in BC cells can affect their migration ability [29]. Herein, we established that mimics of miR-129-1-3p considerably repressed the intracellular Ca^2+^ concentration of MDA-MB-231 cells and reduced the number of Ca^2+^ positive cells. miR-129-1-3p inhibitor dramatically increased the intracellular Ca^2+^ concentrations, as well as Ca^2+-^positive cell numbers expressed. This result indicates that the tumor suppressor effect of miR-129-1-3p is linked to the Ca^2+^ cascade.

We have verified that the Ca^2+^-linked GRIN2D 3′-UTR has a binding site with miR-129-1-3p, which means that miR-129-1-3p can target and negatively regulate the expression of GRIN2D [30]. GRIN2D constitutes a subunit of the glutamate receptor calcium channel. It is worth noting that the glutamate receptor is overexpressed in multiple cancers and plays a vital role in cancer cell growth [31]. Besides, GRIN2D can be used as a critical hub of dysregulated signaling pathways in cancer [32]. Ferguson et al. exhibited that GRIN2D is expressed explicitly in the blood vessels of colorectal cancer and can be used as a specific marker of colorectal cancer angiogenesis [33]. Herein, the expression of GRIN2D mRNA along with protein in MDA-MB-231 cells was detected via qRT-PCR and Western blot assays. The data illustrated that miR-129-1-3p mimics could dramatically reduce the expression of GRIN2D mRNA and Ca^2+^ linked GRIN2D protein in the MDA-MB-231 cells. On the other hand, miR-129-1-3p inhibitor dramatically increased GRIN2D mRNA expression and Ca^2+^ associated protein expression in MDA-MB-231 cells. This finding supports the experimental data of Fluo-3 AM for the determination of Ca^2+^ content in MDA-MB-231 cells. Combined with these results, we confirmed that miR-129-1-3p can inhibit the growth, migration, and infiltration of TNBC cells by dampening the expression of GRIN2D gene and protein, thereby reducing the content of Ca^2+^ in TNBC cells.

The present study enriched the knowledge of the biological functions of miR-129-1-3p and laid a good foundation for the study of the pathogenesis of TNBC. miR-129-1-3p is expected to become an essential target for TNBC treatment with a broad application prospect. For the next research step, we plan to introduce GRIN2D overexpression plasmid to verify the mechanism of miR-129-1-3p repression of TNBC through the interaction mode of miR-129-1-3p and GRIN2D.

## Data Availability

The data generated in this work can be accessed from the corresponding author upon reasonable request.

## Supplementary Materials

Supplementary Materials The details of the reagents and materials used in this study are in Appendix I.
