# Supplementary material for: MicroRNA-129-1-3p Represses the Progression of Triple-Negative Breast Cancer by Targeting the GRIN2D Gene
Source: Biomed Res Int. 2022 Mar 7;2022:1549357. doi: 10.1155/2022/1549357 (PMC8920657; doi:10.1155/2022/1549357)
Supplement: Supplementary Materials — The details of the reagents and materials used in this study are in Appendix I. [file 1549357.f1.docx]

**Supplementary Material**

**Appendix I.** The reagents, materials, or kids used during the experiment.

| **Purpose** | **Reagents, materials, or kids** | **Manufacturer** |
| --- | --- | --- |
| **Reagents and materials** | | |
| Reagents and materials used in the experiment | BCA protein assay kit (P0011) | Beyotime Institute (Shanghai, China) |
|  | Fluo-3 AM (S1056) | Beyotime Institute (Shanghai, China) |
|  | Transwell upper compartment (3422, BD, United States) | Yeasen Biotech (Shanghai, China) |
|  | Artificial basement membrane (356234, BD, United States) | Yeasen Biotech (Shanghai, China) |
|  | 0.5% crystal violet (60506ES60) | Yeasen Biotech (Shanghai, China) |
|  | CCK-8 kit (96992-100TESTS-F) | Sigma (MO, United States) |
| **Quantitative real-time PCR** | | |
| Total RNA isolated | TransZol | ET101-01, Beijing TransGen Biotech |
| cDNA generation from RNA | TransStart Top Green qPCR SuperMix kit | AQ601-04, Beijing TransGen Biotech |
| The contents of GRIN2D mRNA along with GAPDH mRNA | qRT-PCR with the MicroRNAs Quantitation PCR Kit | B300533, Sangon |
| Total miRNA isolated | SanPrep Column MicroRNA Mini-Preps Kit | B518811, Sangon Biological Engineering Technology & Services Co., Ltd.; Shanghai, China |
| cDNA generation from miRNA | MicroRNA First Strand cDNA Synthesis Kit | B300537, Sangon |
| The quantities of miRNA-129-1-3p as well as U6 snRNA | By qRT-PCR with the MicroRNAs Quantitation PCR Kit | B300533, Sangon |
|  |  |  |

The BCA kit (P0011) and Calcium ion fluorescent probe (Fluo-3 AM, S1056) were supplied by. Transwell upper compartment (3422, BD, United States), artificial basement membrane (356234, BD, United States), and 0.5% crystal violet (60506ES60) were supplied by Yeasen Biotech Co., Ltd. (Shanghai, China). Furthermore, CCK-8 assay kit (96992-100TESTS-F) was commercially provided by.
